# Distinct genetic profiles of obesity have different effects on breast cancer risk: leveraging Mendelian randomisation to interrogate causal pathways and identify mediating proteins

**DOI:** 10.1186/s13058-025-02214-3

**Published:** 2026-01-09

**Authors:** Marina Isabel Marchal, Pascal M. Mutie, Tayebeh Azimi, Yaqi Alexandra Deng, Torgny Karlsson, Åsa Johansson

**Affiliations:** Department of Immunology, Genetics and Pathology, Science for Life Laboratory, Uppsala University, 751 85 Uppsala, Sweden

**Keywords:** Breast cancer, Obesity, BMI, Mendelian randomisation, Clustering, Mediation, Proteomic

## Abstract

**Background:**

Obesity is a well-established risk factor for many cancers, but its association with breast cancer remains inconsistent. Obesity is a highly complex and polygenic condition, driven by multiple biological pathways, some of which have been linked to beneficial health effects, defining a metabolically healthy obese phenotype. We hypothesise that this heterogeneity may partly underlie the variable association with breast cancer, where different biological pathways may play divergent roles in disease risk.

**Methods:**

We applied an approach in two parts to explore the heterogeneous effects of high body mass index (BMI) on breast cancer risk. First, we applied Mendelian randomisation (MR)-based clustering on genetic data (FinnGen, GIANT, BCAC) to identify clusters of BMI-increasing SNPs with differential effects on breast cancer, and the cluster-specific MR results were replicated in the UK Biobank. Second, we integrated proteomic data to estimate the cluster-specific effects of BMI on protein levels and the effect of these proteins on breast cancer risk, using two-sample MR. Mediation analyses were then carried out using the product-of-coefficient method*.*

**Results:**

We identified three clusters of SNPs associated with increased BMI while having differential effects on breast cancer risk: one risk-increasing (inverse variance weighted MR estimate: 1.27 [1.10, 1.44]), one medium protective (− 0.75 [− 0.82, − 0.68]), and one highly protective (− 1.92 [− 2.21, − 1.62]), comprising 24, 73, and 7 SNPs respectively, with similar magnitude of effects in the UK Biobank. In the two-sample MR, each cluster was associated with many proteins (70, 369 and 44 respectively, FDR q-value < 0.05), and the mediation analysis identified that three proteins significantly mediate cluster-specific effects on breast cancer risk. Among these proteins, MET is known to be involved in breast cancer, while little is known about the roles of CPM and CST6.

**Conclusions:**

These findings suggest that the effect of obesity on breast cancer is mediated through multiple distinct biological pathways. The identified proteins provide a first insight into the mechanisms underlying these pathways. With further investigation, these results could provide a basis for developing personalised treatment strategies.

**Supplementary Information:**

The online version contains supplementary material available at 10.1186/s13058-025-02214-3.

## Background

Breast cancer is the leading cause of cancer death in women, and fourth across all sexes [1]. With an estimated 2.3 million new cases in 2022, it is the most frequently diagnosed type of cancer worldwide. By 2050, breast cancer incidence is predicted to reach 3.2 million per year, and the number of deaths caused by breast cancer to be 1.1 million [1]. Obesity has been highlighted as a driver and major risk factor for breast cancer [2, 3] Several different mechanisms related to obesity could contribute to breast cancer development and worsen prognosis. For example, obesity is known to create a pro-inflammatory environment, stimulating carcinogenesis [4]. In addition, adipose tissue plays an important role in the secretion of molecules relevant to endocrine signalling. As the amount of tissue available increases, the levels of generated hormones and peptides are dysregulated, further contributing to the creation of an environment favourable to tumour development [5, 6].

However, different study designs have produced conflicting results, with prospective studies in premenopausal women as well as Mendelian randomisation (MR) studies typically showing an association between obesity and lower rates of breast cancer diagnoses [3]. MR is a method in observational epidemiology based on the concept of randomisation of the genotype, which has gained large popularity [7]. Interestingly, in contrast to most observational studies, these studies found an inverse relationship with breast cancer risk as body mass index (BMI) increased [3, 8–10]. In traditional MR, the causal estimate between exposure and outcome is calculated for each genetic variant, also known as an instrumental variable (IV). When an IV’s causal estimate differs too much from others, it could be a sign of heterogeneity or a violation of certain MR assumptions, and therefore often deemed to be invalid [11]. This, however, implies all IVs mediate the same effect on the exposure, ignoring the possibility of different genetic variants acting on the exposure via different biological mechanisms. If not properly accounted for, multiple effector pathways could result in weak causal estimates or inconsistent results across different MR studies [12, 13]. To overcome this limitation, novel methods for MR analyses, such as MR Clust, have been developed. The MR Clust algorithm groups IVs with similar causal estimates both on the exposure and the outcome into clusters. Given this similarity, they are likely to affect the exposure via a shared biological pathway representative of pathology [12]. In type 2 diabetes for example, BMI-increasing variants have been found to have differential effects on the risk of developing the disease via different mechanisms [14].

To our knowledge, no study so far has conducted MR-based clustering to investigate the effect of obesity on breast cancer. Similarly, as observed in type 2 diabetes, the causal effect of weight gain on breast cancer might vary based on the underlying reasons and genetic variants for obesity, causing the conflicting results observed. Other factors such as low parity, late first pregnancy, early menarche or late menopause are likely to play a role as well [15]. Menopausal status for example influences oestrogen production, with the ovaries being the primary source of oestrogen pre-menopause, not the adipose tissue. In this case, the adipose tissue can sequester large parts of oestradiol, lowering levels of circulating oestrogen. Another potential mechanism lowering oestrogen levels could be an increased liver clearance of oestradiol in obese women pre-menopause [16, 17]. More generally, different types of oestrogen such as oestrone, oestradiol, or the ratio between the two, have been shown to have different roles in breast cancer development, and have different expression profiles according to obesity and menopause status [18]. In addition, while obesity is generally a risk factor for many diseases, it is not always concordant with worse health. A metabolically healthy obese phenotype associated with lowered systolic blood pressure and type 2 diabetes risk has been characterised, showing the heterogeneity of this trait [19, 20]. Given these variations in health outcomes associated with a same trait, the high polygenicity of obesity, and the complexity of the relationship between obesity and breast cancer, we aimed at elucidating some of the heterogeneity in results between studies by applying MR-based clustering to large-scale multi-omics. In a first step, clusters of genetic variants were identified using MR Clust on BMI and breast cancer genome wide association studies (GWAS) summary statistics. In a second step, to follow up on potential cluster-specific pathological mechanisms, we looked at proteins mediating cluster effects on breast cancer.

## Methods

### Study design

**Fig. 1:**
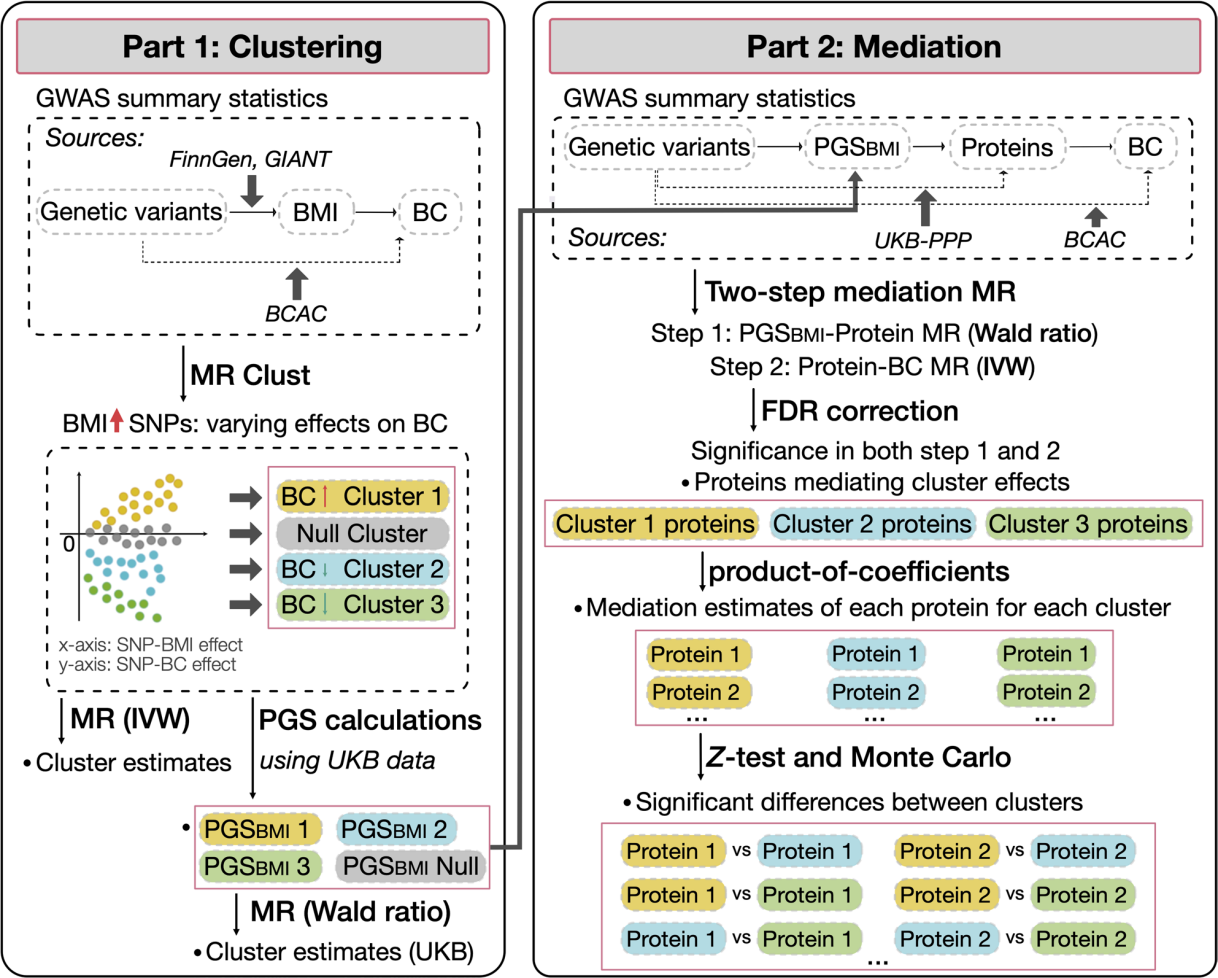
Overview of the study. BC: breast cancer; BCAC: breast cancer association consortium; BMI: body mass index; GWAS: genome-wide association study; IVW: inverse-variance weighted; MR: Mendelian randomisation; PGS_BMI_: polygenic score weighted on BMI; PPP: Pharma Proteomics Project; SNP: single nucleotide polymorphism; UKB: UK Biobank

An overview of the study design is presented in Fig. 1. In the first part we performed MR-based clustering using GWAS summary statistics for BMI and breast cancer to identify clusters of single nucleotide polymorphisms (SNPs) associated with increased effect on BMI, but with differential effects on breast cancer risk. These clusters were then used to compute cluster-specific two-sample MR estimates. To corroborate the clusters in an independent cohort, we computed cluster-specific polygenic scores (PGS) for BMI (PGS_BMI_) in the UK Biobank and estimated the causal effect of BMI clusters on breast cancer risk using the Wald ratio method in a one-sample MR framework.

The second part aimed at identifying proteins that mediate each cluster’s effects on breast cancer risk upon increased BMI, performing two-step mediation MR. In the first step of the mediation MR, the effect of BMI on protein levels was estimated by using the Wald ratio method in a two-sample MR framework in two separate subsamples of the UK Biobank samples. In the second step of the mediation MR, the effect of protein levels on breast cancer was estimated by performing inverse variance weighted (IVW) two-sample MR on GWAS summary statistics from the UK Biobank Pharma Proteomics Project (UKB-PPP) for proteins and the Breast Cancer Association Consortium (BCAC) for breast cancer. Mediation estimates and proportions were calculated for proteins identified as significant mediators after false discovery rate (FDR) correction. Pairwise differences between clusters were tested using the *Z*-test with standard errors estimated by the delta method, followed up by confidence intervals (CIs) estimated by Monte Carlo simulations.

### GWAS summary statistics data for clustering

For the clustering analysis, we meta-analysed BMI GWAS summary statistics meta-analysis results from FinnGen (release 9) [21] and the GIANT consortium (2018 release) [22]. Data for 266,130 and 322,154 individuals of European ancestry from FinnGen and GIANT, respectively, were included in this analysis. They were meta-analysed using fixed-effect IVW method, resulting in 513 independent SNPs significantly associated with BMI (*p* < 5 $\times$ 10^−8^) after clumping using PLINK v1.9 [23], with window and linkage disequilibrium threshold parameters of kb = 500 and *r*^2^ = 0.001, respectively. The strict *r*^2^ was used to ensure independence among SNPs in the MR analyses.

For breast cancer, we used GWAS summary statistics combining studies from the BCAC as well as other GWAS studies, containing 10.8 million SNPs from 133,384 cases and 113,789 controls of European ancestry [24]. These were also used for the second step of the mediation MR. To further evaluate oestrogen receptor (ER)-subtype–specific effects, we incorporated additional GWAS summary statistics derived from the OncoArray project (covering 68 BCAC cohorts), the Collaborative Oncological Gene–Environment Study (iCOGS), and 11 other breast cancer GWAS studies. These summary statistics include 11.7 million SNPs from 272, 977 cases and 105,974 controls of European ancestry, of which 69,501 are ER-positive cases and 21,468 are ER-negative cases [25].

### UK Biobank data

The UK Biobank contains large amounts of health-related individual-level data for about 500,000 participants, combined from data collection at recruitment, health records, national hospital and cancer registers. All participants were genotyped for 800,000 SNPs, a number increased to 90 million SNPs by imputation of untyped variants. The cohort is described in more detail by Bycroft and colleagues [26]. We selected unrelated participants of European ancestry according to self-report as of white British descent, classification as Caucasian by principal component analysis, genetic relatedness pairing (data fields 21000, 22006 and 22011, respectively); and excluded according to pairwise relatedness based on kinship data (estimated genetic relationship > 0.044), poor call rate (< 95%), high heterozygosity or sex discordance (data fields 22010 and 22001, respectively). After excluding those lacking BMI data, this resulted in a sample size of 361,748 individuals.

Measurements of plasma protein levels are available for 54,219 individuals through the UKB-PPP initiative. Of these, 46,595 were selected at random, 6,376 were selected by the UKB-PPP consortium members, and 1,268 were selected from the COVID-19 repeat-imaging study. Plasma protein levels for a total of 2,923 proteins were measured using the OLINK Explore 3072 platform, followed by quality control and batch normalisation [27]. After excluding one protein with measurements below detection limit for more than 90% of participants, 2,922 were available for downstream analyses. Keeping only unrelated individuals of European ancestry, protein measurements for 38,562 participants were available, of which 20,721 females were included in the analyses with individual level proteomic data.

Breast cancer cases in the UK biobank were defined using data from cancer registries on cancer type, provided in ICD-9 or ICD-10 coding (data fields 40013 and 40006, respectively), as well as hospital in-patient data (data field 41202, 41203, 41204 and 41205) and cause of death (data fields 40001 and 40002). Self-reported data on cancer type provided during verbal interviews at the assessment centres was also utilised (data field 20,001). After filtering for European ancestry, data on breast cancer occurrence was available for 194,682 women, of which 16,496 developed the disease.

In analyses where individual level data was used, several covariates were considered. Age (data field 21022), age squared, sex (22001), genetic array (22000), UK Biobank centre (54), and principal components 1–20 (22009.0.1–20) were extracted to be included as covariates in the different linear and logistic models. Inclusion in the UKB-PPP cohort (30903), and batch number (via plate number, 30901) were included as covariates where relevant in models involving proteomic data. For the sensitivity analyses, we stratified participants into pre- and post-menopausal groups using self-reported data (2724), excluding all women who were unsure about their menopausal status.

For the second step of the mediation, we selected IVs for the MR from the GWAS summary statistics on plasma protein levels, obtained from analysis in the UKB-PPP cohort described previously [27].

### Mendelian randomisation clustering

Prior to clustering, data was prepared in R (v4.3.2) [28] using the TwoSampleMR package (v0.5.8) [29]. The 513 independent BMI-associated SNPs from our meta-analysis were checked for duplicates, non-biallelic alleles and being palindromic, as part of the harmonisation with breast cancer summary statistics, formatting BMI as the exposure and breast cancer as the outcome. Palindromic SNPs that could not be resolved by harmonisation and SNPs with ambiguities in strand orientation were removed. Steiger filtering was applied, additionally removing all SNPs that did not significantly explain more variation in BMI than in breast cancer risk (p < 0.05), resulting in a total of 464 SNPs that were submitted to clustering.

Clustering of SNPs with similar causal estimates for BMI and breast cancer was performed using the MR Clust package (v0.1.0). This soft-clustering method based on the expectation–maximization algorithm, was notably chosen for its inclusion of null and junk clusters, prioritising strong associations [12]. Cluster estimates (IVW MR estimates) were obtained by performing Two-sample MR for each cluster, using the TwoSampleMR package. Further sensitivity analyses were conducted to evaluate the cluster-specific effects on ER-positive and ER-negative breast cancer, in addition to the overall breast cancer risk that was used during the clustering.

### Polygenic score calculations for BMI

To reduce the risk of weak instrument bias and account for the limited number of SNPs in some clusters, we constructed cluster-specific PGSs by aggregating all available BMI-associated variants within each cluster. These PGSs were used as joint instruments in the MR analyses. This approach strengthens instrument validity by increasing the explained variance in BMI and provides more stable causal estimates, particularly in clusters with few individual SNPs. Four independent weighted allele scores on BMI (PGS_BMI_), corresponding to the four clusters, were computed (PLINK, v1.9) [30] for each individual in the UK Biobank, using individual-level genetic data. Weights for each SNP were taken from the FinnGen and GIANT meta-analysis described above.

### UK Biobank MR analyses for cluster-specific BMI effects on breast cancer

Logistic regression models were run with the PGS_BMI_ of each cluster as predictor of breast cancer for women in the UK Biobank. Linear regression models were run with the cluster-specific PGS_BMI_ as predictor of BMI. BMI was rank-based inverse normal transformed similarly to the original GWAS summary statistics from FinnGen and GIANT used to extract weights for the PGS_BMI_. The MR estimates for each cluster were calculated using the Wald method [31, 32].

### Mediation MR step 1: two-sample MR analysis to estimate cluster specific BMI effects on proteins

Similarly to the above, linear models were run to estimate the effect of cluster-specific PGS_BMI_ but this time on protein levels in the female UKB-PPP sample (*n* = 20,721). Here, the effect of the PGS_BMI_ on BMI was estimated in an independent sub-sample (*n* = 341,027) not containing the participants included in the UKB-PPP cohort, to enable a two-sample MR setting within the UK Biobank cohort. The corresponding effects of BMI on protein levels were estimated using the Wald method [31, 32]. Benjamini–Hochberg’s FDR correction was applied to identify proteins differentially expressed for each cluster in the MR analysis. Further sensitivity analyses were conducted to evaluate the cluster-specific PGS_BMI_ in the post- and pre-menopausal strata, and a Z-test was used to assess whether the cluster-specific effects differed between the two strata.

### Mediation MR step 2: two-sample MR analysis to estimate protein effects on breast cancer

Using the previously described GWAS summary statistics from the UKB-PPP and BCAC, two-sample MR was performed to obtain estimated effects of proteins on breast cancer. Prior to two-sample MR, proteins were filtered to retain only independent and genome-wide significant (*p* < 1.7 × 10^−11^) proteins with at least three independent SNPs (minimum one cis-regulatory) to ensure safeguarding of MR assumptions, resulting in a total of 1,594 proteins. Two-sample MR was performed using the TwoSampleMR package (v0.5.8) [29], retaining the inverse-variance weighted (IVW) estimate. Horizontal pleiotropy and heterogeneity (*p* < 0.05) were evaluated using the MR-Egger intercept test and Cochran’s Q statistic, respectively.

### Identification of proteins significantly mediating the effect of BMI on breast cancer

Benjamini–Hochberg’s FDR correction was applied to the *p*-values of both MR steps described above, i.e., BMI on proteins and proteins on breast cancer. For proteins resulting to be significant in both steps, the mediated effect was estimated using the product-of-coefficients method as the product of the two total effects from these two univariable MR analyses. Estimates and CIs were calculated using the Monte Carlo method implemented in the RMediation package [33]. This approach provides robust confidence interval estimates by simulating the distribution of the product of coefficients. It is particularly well-suited for addressing deviations from normality when applying the product-of-coefficients method to estimate mediated effects. The proportion of effect mediated was calculated by dividing the estimate of the mediated effect (the product of the two total effects) by the total effect of the respective cluster as determined by the two-sample MR IVW estimate for BMI on breast cancer, as described above. Note, however, that the mediated proportions estimated here are only approximate. This is due to the non-collapsibility of the odds ratio (OR), which is used as measure of the breast cancer risk. The estimated OR formally depends on the specific set of covariates used in the logistic modelling, wherefore also the mediated proportions are estimated for the specific covariate set used in our analysis.

The *Z*-test was adopted to test for differences in mediating effects between different clusters of BMI-associated SNPs, for individual proteins. Since the second MR estimate (protein on breast cancer) is identical across all clusters for a given protein, the estimate of the difference in mediating effects can be rearranged into a product of the effect of protein on breast cancer (second MR step) and the difference in cluster-specific BMI effects on the given protein (first MR step). This difference in MR estimates for a certain protein P between two clusters (A and B), denoted as${\widehat{\beta }}_{\mathrm{diff}}$, is simply computed as$\hat{\beta }_{{{\mathrm{diff}}}} = \hat{\beta }_{{{\text{cluster A}} \to {\mathrm{P}}}} - \hat{\beta }_{{{\text{cluster B}} \to {\mathrm{P}}}}$. The corresponding standard error of this difference, ${\widehat{\sigma }}_{\mathrm{diff}}$ is estimated as: $\hat{\sigma }_{{{\mathrm{diff}}}} = \sqrt {\hat{\sigma }_{{{\text{cluster A}} \to {\mathrm{P}}}}^{2} + \hat{\sigma }_{{{\text{cluster B}} \to {\mathrm{P}}}}^{2} }$. Applying the delta method for the product of two random variables, a proximal estimate of the standard error ($\widehat{\sigma })$ of the difference in mediating effects is then given by

$$ \hat{\sigma } = \sqrt {\hat{\beta }_{{{\mathrm{diff}}}}^{2} \cdot \hat{\sigma }_{{{\mathrm{P}} \to {\mathrm{BC}}}}^{2} + \hat{\beta }_{{{\mathrm{P}} \to {\mathrm{BC}}}}^{2} \cdot \hat{\sigma }_{{{\mathrm{diff}}}}^{2} } , $$

where, $\hat{\beta }_{{{\mathrm{P}} \to {\mathrm{BC}}}}$ and $\hat{\sigma }_{{{\mathrm{P}} \to {\mathrm{BC}}}}$ denote the MR estimate and standard error, respectively, of the protein P on breast cancer risk. To improve the accuracy of the 95% confidence intervals, particularly for estimates near the threshold of statistical significance, we applied a Monte Carlo procedure. This method resamples the distribution of the MR estimates to empirically derive confidence intervals, offering a more robust alternative to the analytical approximation used above.

### Code availability

Full code for all analyses in this study will be made available on GitHub (https://github.com/AJResearchGroup).

## Results

### Four clusters are differentially associated with obesity and breast cancer risk

We meta-analysed GWAS summary statistics data for BMI from the FinnGen (*n* = 266,130) and GIANT consortium (*n* = 322,154) and identified 513 independent SNPs (*p* < 5 × 10^−8^) that were selected as instrumental variables for downstream MR analysis. After aligning these with GWAS summary statistics for breast cancer from the BCAC (cases = 133,384; controls = 113,789) and further quality control, 464 SNPs remained for clustering analysis (Supplementary Table S1).

**Fig. 2:**
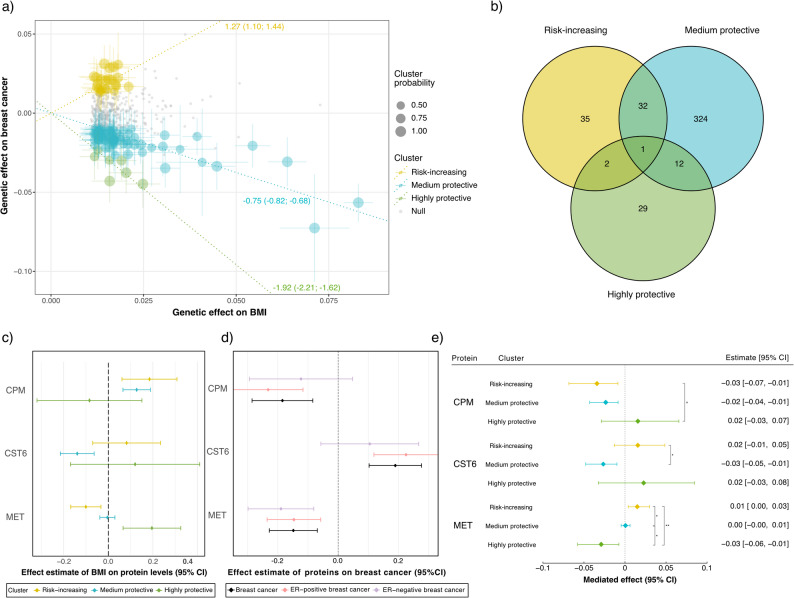
**a** Genetic variants plotted based on their effect on BMI and breast cancer. Circle size represents the probability of variants to belong to their respective cluster, error bars their 95% CIs. Annotated regression lines indicate the IVW estimate (log[OR]) and 95% CIs for each cluster by two-sample MR. Gray dots represent variants belonging to the null cluster and appear to have no effect on breast cancer. **b** Overlap between clusters of proteins significantly (FDR < 0.05) affected by PGS_BMI_ according to two-sample MR analysis in the UK Biobank, as estimated by the Wald method. **c** Effect of PGS_BMI_ on protein levels per cluster, for the three proteins that were significant in both MR steps. **d** Effect of the same three proteins on any type of breast cancer, ER-positive and ER-negative breast cancer risk (log[OR]), as estimated by two-sample IVW MR. **e** Mediated effect by proteins in the obesity-breast cancer relationship per cluster, and significance of the difference between clusters. Significant differences in mediated effects per protein between clusters were tested by Sobel and Monte Carlo-estimated CIs, significance marked as **: *p* < 0.01, *: *p* < 0.05, and °: only Monte Carlo-estimated CIs significant. Negative mediation estimates indicate a protective effect. BMI: body mass index; CI: confidence interval; IVW: inverse-variance weighted; MR: Mendelian randomisation; OR: odds ratio; PGS_BMI_: polygenic score weighted on BMI

Using MR Clust, four clusters of genetic variants associated with increased BMI were identified to have differential effects on breast cancer (Fig. 2a). One cluster, assigned as null cluster, contained 360 SNPs which appeared to have no effect on breast cancer risk. One cluster with a positive effect, from here on referred to as the risk-increasing cluster, was constituted of 24 SNPs. Two clusters showed varying negative i.e. protective effects of obesity on breast cancer risk with 73 and 7 SNPs, from here on referred to as the medium protective and highly protective clusters, respectively. Cluster-specific effect estimates were calculated by two-sample MR, retaining the IVW estimate (Fig. 2a). Similar MR estimates were observed for ER-positive breast cancer, whereas the results for ER-negative breast cancer were slightly less pronounced and not all the three clusters reached statistical significance, possibly due to the smaller sample size (Table 1).

**Table 1:** MR estimates (log[OR]) for the effect of BMI on any type of breast cancer

	Number of SNPs	Cluster mean	Two-sample IVW MR estimates* (95% CI)			UK Biobank Wald ratio MR estimate (95% CI)
		Any type of breast cancer	Any type of breast cancer	ER-positive breast cancer	ER-negative breast cancer	Any type of breast cancer
Risk-increasing cluster	24	0.98	1.27 (1.10, 1.44)	1.22 (0.99, 1.45)	0.90 (0.43, 1.37)	0.97 (0.61, 1.31)
Medium protective cluster	73	− 0.61	− 0.75 (− 0.82, − 0.68)	− 0.73 (− 0.82, − 0.64)	− 0.84 (− 0.99, − 0.69)	− 0.43 (− 0.60, − 0.25)
Highly protective cluster	7	− 1.74	− 1.92 (− 2.21, − 1.62)	− 2.10 (− 2.46, − 1.74)	− 1.42 (− 1.97, − 0.86)	− 1.53 (− 2.19, − 0.86)
Null cluster	360	0.023	0.04 (− 0.01, 0.08)	0.05 (0.00, 0.10)	0.00 (− 0.08, 0.09)	0.01 (− 0.10, 0.12)

^*^Two − sample IVW MR estimates (95% CI) using FinnGen + GIANT BMI estimates and BCAC breast cancer estimates for any breast cancer and Oncoarray + iCOGS + GWAS estimates for ER-positive and ER-negative breast cancer. ER-positive and ER-negative breast cancer for the four clusters; CI: confidence interval; IVW: inverse-variance weighted; MR: Mendelian randomisation; OR: odds ratio; SNP: single nucleotide polymorphism.

### All four clusters can be reproduced in an independent cohort, the UK Biobank

When gaining weight, different underlying genetic variants for obesity are likely to affect the risk of developing breast cancer in different ways, by acting through different pathways. We wanted to test this hypothesis by estimating the effects of these clusters in an independent cohort. To gain power, we computed cluster-specific PGS_BMI_ for individuals of European ancestry in the UK Biobank (*n* = 361,887), weighted by the effect estimates for the respective SNPs extracted from the GIANT and FinnGen meta-analysis.

The association of breast cancer predicted by PGS_BMI_ was estimated by logistic regression and used to calculate one-sample Wald ratio MR estimates in the UK Biobank. These estimates (log[OR]) were 0.97 (0.61, 1.31; 95% CI) for the risk-increasing cluster, − 0.43 (− 0.60, − 0.25; 95% CI) for the medium protective cluster, − 1.53 (− 2.19, − 0.86; 95% CI) for the highly protective cluster, and 0.01 (− 0.10, 0.12; 95% CI) for the null cluster. Overall, the effect estimates were recapitulated in the UK Biobank one-sample MR for all clusters (Table 1). Nevertheless, the effect sizes appear to be slightly weaker in the UK Biobank, especially for the medium protective cluster, which agrees with the combined effect of winner’s curse and overfitting during clustering in the discovery cohorts.

### A total 435 proteins are affected by at least one BMI cluster

As the clusters have different effects on breast cancer risk, it is likely that they represent different pathogenic or protective mechanisms. To investigate some of these potential pathways further, two-step mediation MR was performed with proteins as potential mediators of the effect of BMI on breast cancer risk.

The first step of the mediation analysis consisted of a two-sample MR of two independent sub-samples within the UK Biobank of which the first subset included all participants except the 38,562 with proteins measurements. For each non-null cluster, linear models were run to estimate the effect of the respective cluster-specific PGS_BMI_ on BMI and protein levels for 2,922 proteins. The linear model estimates were then used to calculate MR estimates using the Wald ratio method. After FDR correction, 435 proteins were identified to be significantly affected by at least one cluster-specific PGS_BMI_, with 70, 369, and 44 affected by the risk increasing, the medium, and the highly protective cluster, respectively (Fig. 2b and Supplementary Table S2). The larger number of proteins associated with the medium protective cluster agrees with a larger number of SNPs belonging to that cluster, resulting in higher power to detect an association with protein measurements (Table 1). We also calculated MR estimates in two strata of pre- and post-menopausal women (*n* = 4,015 and *n* = 12,171, respectively). Although the estimates fluctuated slightly between the full cohort and the two strata (Supplementary Table S2), none of the proteins showed significant differences between the pre- and post-menopausal groups (Z-test, FDR > 0.05). Therefore, menopausal-stratified analyses were not considered further.

### A total 21 proteins are associated with breast cancer risk

In the second step of the mediation analysis, we ran a two-sample MR analysis, estimating the effect of 1,594 proteins (selection criteria outlined in the methods section) from the UK Biobank on breast cancer in the BCAC. The IVW estimates were retained and FDR correction applied to the p-values, revealing 21 proteins (Table 2 and Supplementary Table S3) to significantly affect breast cancer risk (FDR < 0.05). We also estimated the IVW effects separately for ER-positive and ER-negative breast cancers. The results for ER-positive disease were largely consistent with those observed in the overall breast cancer cohort. In contrast, while some estimates for ER-negative breast cancer aligned with those for ER-positive disease, others showed no evidence of association (Table 2).

**Table 2:** Proteins with significant (FDR < 0.05) IVW MR estimate on any type

Protein	Full Name	IVW MR estimates (95% CI)		
		Any breast cancer	ER positive	ER negative
PTPRM	Receptor-type tyrosine-protein phosphatase mu	− 0.106 (− 0.138, − 0.073)	− 0.112 (− 0.154, − 0.070)	− 0.103 (− 0.164, − 0.042)
MECR	Enoyl-[acyl-carrier-protein] reductase, mitochondrial	− 0.609 (− 0.818, − 0.400)	− 0.687 (− 0.946, − 0.428)	− 0.019 (− 0.412, 0.374)
EPHA4	Ephrin type-A receptor 4	− 0.118 (− 0.162, − 0.073)	− 0.120 (− 0.180, − 0.060)	− 0.099 (− 0.177, − 0.021)
ICAM5	Intercellular adhesion molecule 5	− 0.095 (− 0.131, − 0.060)	− 0.112 (− 0.156, − 0.068)	− 0.071 (− 0.138, − 0.004)
OMG	Oligodendrocyte-myelin glycoprotein	− 0.368 (− 0.521, − 0.216)	− 0.360 (− 0.549, − 0.172)	− 0.573 (− 0.866, − 0.281)
ENG	Endoglin	− 0.116 (− 0.167, − 0.065)	− 0.123 (− 0.187, − 0.059)	− 0.142 (− 0.217, − 0.067)
LRRC37A2	Leucine-rich repeat-containing protein 37A2	− 0.061 (− 0.087, − 0.034)	− 0.060 (− 0.095, − 0.026)	− 0.042 (− 0.081, − 0.002)
TMPRSS15	Enteropeptidase	− 0.188 (− 0.271, − 0.106)	− 0.184 (− 0.279, − 0.089)	− 0.108 (− 0.222, 0.005)
PTPRK	Receptor-type tyrosine-protein phosphatase kappa	− 0.372 (− 0.540, − 0.203)	− 0.369 (− 0.628, − 0.109)	− 0.423 (− 0.652, − 0.193)
CST6	Cystatin-M	0.190 (0.103, 0.277)	0.225 (0.119, 0.332)	0.105 (− 0.057, 0.267)
TACC3	Transforming acidic coiled-coil-containing protein 3	− 0.226 (− 0.336, − 0.116)	− 0.209 (− 0.410, − 0.009)	− 0.327 (− 0.665, 0.010)
MTIF3	Translation initiation factor IF-3, mitochondrial	− 0.529 (− 0.802, − 0.255)	− 0.573 (− 0.907, − 0.238)	− 0.276 (− 0.692, 0.140)
TNFRSF17	Tumour necrosis factor receptor superfamily member 17	− 0.138 (− 0.209, − 0.067)	− 0.171 (− 0.262, − 0.079)	0.017 (− 0.122, 0.155)
CEP43	Centrosomal protein 43	− 0.242 (− 0.369, − 0.116)	− 0.247 (− 0.403, − 0.091)	− 0.170 (− 0.409, 0.068)
GATD3	Glutamine amidotransferase-like class 1 domain-containing protein 3B, mitochondrial	− 0.508 (− 0.774, − 0.243)	− 0.569 (− 0.866, − 0.273)	− 0.068 (− 0.404, 0.269)
MET	Hepatocyte growth factor receptor	− 0.148 (− 0.228, − 0.069)	− 0.147 (− 0.236, − 0.058)	− 0.190 (− 0.299, − 0.081)
CPM	Carboxypeptidase M	− 0.185 (− 0.286, − 0.084)	− 0.232 (− 0.348, − 0.116)	− 0.123 (− 0.294, 0.048)
DCBLD2	Discoidin, CUB and LCCL domain-containing protein 2	− 0.169 (− 0.262, − 0.076)	− 0.157 (− 0.260, − 0.054)	− 0.238 (− 0.444, − 0.032)
LIFR	Leukaemia inhibitory factor receptor	− 0.140 (− 0.218, − 0.062)	− 0.160 (− 0.264, − 0.056)	− 0.123 (− 0.232, − 0.013)
MEGF11	Multiple epidermal growth factor-like domains protein 11	0.223 (0.098, 0.347)	0.236 (0.081, 0.392)	0.035 (− 0.200, 0.270)
SLC9A3R1	Na( +)/H( +) exchange regulatory cofactor NHE-RF1	− 0.372 (− 0.585, − 0.159)	− 0.410 (− 0.673, − 0.146)	− 0.013 (− 0.415, 0.389)

ER-positive and ER-negative breast cancer risk. CI: confidence interval; FDR: false discovery rate; IVW: inverse-variance weighted; MR: Mendelian randomisation.

### Three proteins potentially mediating the effect of obesity on breast cancer are differentially expressed between clusters

Out of these 21 proteins (Table 2), three proteins were also significantly influenced by at least one of the non-null cluster-specific PGS_BMI_ and thereby identified as potential mediators: carboxypeptidase M (CPM), cystatin M (CST6), and hepatocyte growth factor receptor (MET). Not surprisingly, all three proteins showed significant (*p* < 0.05) mediation effects for at least one of the clusters (Fig. 2e).

In the first step of the mediation MR (cluster-specific BMI effects on proteins), the risk-increasing cluster-specific PGS_BMI_ decreased MET, while the most protective cluster-specific PGS_BMI_ was associated with increased MET (Fig. 2c). The second mediation MR step (protein effect on breast cancer) revealed that increased MET decreases the risk of breast cancer (Fig. 2d). This implies that a decrease in MET levels caused by the risk-increasing cluster specific PGS_BMI_ is causing an increased risk of breast cancer. Oppositely, the highly protective cluster specific PGS_BMI_ increased MET, implying that this cluster notably decreases the risk for breast cancer by upregulating MET. From our analyses, MET mediated 1.18% of breast cancer risk associated with the risk-increasing cluster and 1.51% of the protective effect of the highly protective cluster. No effects of the medium protective cluster appeared to be mediated by MET. Looking at the overall mediation estimates (Fig. 2e), all effects of MET were significantly different from each other between the clusters.

CST6 significantly decreased as PGS_BMI_ increased in the medium protective cluster but was not significantly affected by the other clusters. Since CST6 appears to increase the risk of breast cancer, this implies the medium protective cluster should reduce breast cancer risk notably by decreasing CST6 levels. This effect was found to be significantly different from the effect CST6 mediates for the risk-increasing cluster, but not from the highly protective cluster. We cannot, however, rule out the possibility that the lack of effect by the highly protective cluster is due to lack of statistical power, as the medium protective cluster comprises substantially more SNPs (Table 1). CPM, on the other hand, significantly increased in both the risk-increasing and medium protective clusters as PGS_BMI_ increased, while mediating a decrease of breast cancer risk. This suggests both clusters appear to decrease breast cancer risk via increased CPM levels. Here the Monte Carlo CIs for the mediated effects differed significantly between the risk-increasing and highly protective cluster.

## Discussion

In this study, we applied MR clustering to large-scale genetic data from FinnGen, GIANT, BCAC, and the UK Biobank to identify distinct causal pathways linking higher BMI with breast cancer risk. Our analysis revealed four clusters of genetic variants positively associated with increased BMI, while having different causal effects on breast cancer: one risk-increasing, one medium protective, one highly protective and one null cluster of SNPs with no evidence of causal effect on breast cancer. In subsequent mediation analyses, we identified proteins mediating the distinct effects the different BMI clusters on breast cancer. CPM mediated effects of the risk-increasing and medium protective clusters, CST6 mediated effects of the medium protective cluster alone, and MET mediated effects of the risk-increasing and highly protective clusters, with varying effect directions. When stratifying by ER-positive and ER-negative breast cancer, we observed similar effect sizes and directions for the significant proteins compared with the overall breast cancer analysis. The small, attenuated associations in the ER-negative stratum may be attributable to the smaller sample size.

MET is a hepatocyte growth factor (HGF) receptor embedded in the extra-cellular matrix. It autophosphorylates upon binding of its ligand, HGF, activating downstream signalling. In normal physiology, this signalling is involved in wound healing, tissue repair and regeneration notably by stimulating survival, proliferation and migration of stem cells [34–36]. In cancer cells however, these functions can promote invasion and metastasis, and overexpression of MET has been associated with poor survival [37]. In our analysis, the two-sample MR showed that individuals with a genetic predisposition to express more MET over their lifetime have a lower risk of breast cancer. At the same time, variants in the risk-increasing cluster caused lower MET levels, so it appears that this increased risk conferred by the genetic variants contained in that cluster mediate that risk by lower expression of MET from birth. In contrast, the highly protective cluster caused higher levels of MET, indicating it likely mediates part of its protective effect by having higher MET expression. While this might appear counterintuitive at first, it must be kept in mind that we are looking at genetically predicted expression of MET and therefore life-long patterns of expression, not just in disease. In cancer, HGF and MET increase each other through an autocrine loop [38]. It is likely that the produced HGF in turn not only acts on MET in tumorous cells, but also on healthy cells that do not contribute to tumour progression. We therefore hypothesise that this constitutively increased expression of MET on healthy cells protects individuals, by competing for HGF-binding with MET on tumorous cells.

CPM is an enzyme involved in the cleaving of C-terminal amino acids from peptides and proteins. In normal physiology, it seems to be involved in adipogenesis [39] and hematopoietic differentiation [40, 41], however, evidence of potential involvement in breast cancer is more sporadic. One study found the *Cpm* gene to be rearranged and its expression to be de-regulated in radiation-transformed epithelial breast cell lines, an in vitro model of breast cancer [42]. Another study indicates CPM could be elevated in invasive ductal cell carcinoma of the breast, yet with no indication of significance [43]. Our analysis indicated that higher levels of CPM decrease the risk for breast cancer, while genetic variants in both the risk-increasing and the medium-protective cluster appear to increase CPM. This may appear contradicting, as we hope to identify different disease mechanisms for distinct clusters, however, even if clusters have distinct overall effects on breast cancer it could still be possible that they share certain pathways.

The protein CST6 plays a role in tissue architecture as an inhibitor of cysteine proteases which remodel tissues by proteolysis. In the literature *CST6* expression often appears to be downregulated in breast cancer and other tumours via methylation of its promoter, categorising it as a tumour suppressor gene [44]. This downregulation appears to facilitate tumour progression and metastasis [45]. Some reports, however, highlight a positive relationship with breast tumour size and progression [46, 47]. In our analysis, increased CST6 levels were shown to increase breast cancer risk, with decreased CST6 in the medium protective cluster. This implies the medium protective cluster partly mediates its protective effects by downregulating CST6. These findings increase the evidence for a more complex role of CST6 in breast cancer as has been highlighted before and warrants further investigation [48, 49].

Most previous MR studies that look at the effect of obesity on breast cancer have only employed standard unidirectional MR [3, 8, 10], with some also using weighted allele scores [9]. To our knowledge, this study is the first of its kind to apply MR clustering to investigate differential causal mechanisms of obesity on breast cancer. Findings are based on large-scale population data from several independent registries, including FinnGen, the GIANT and BCAC consortia, as well the UK Biobank and their recently released proteomics data.

While the UKB-PPP data is a major advance to gain disease insights via proteomics, it is still not fully representative of the human proteome, and from the 2, 923 represented in the UK Biobank proteomics release, about half were used in this analysis to ensure MR assumptions can be met. The actual proportion of the effect mediated by the identified proteins are also rather small, highlighting the need of continued investigation to further characterise the pathways they are involved in. In addition, as has been reported before, the population included in the UK Biobank is not entirely representative of the population in the UK as it suffers from a healthy selection bias [50], and with the initial recruitment age range set from 40 to 69 years, it is not surprising that only 23% of women are pre-menopausal [51, 52]. This is of relevance since the effect of obesity on breast cancer risk differs before and after menopause [15]. Unfortunately, there is a lack of large-scale cohorts stratifying for pre- and post-menopausal breast cancer, which limits research into the impact of this factor. The UK Biobank also includes too few pre-menopausal breast cancer incidences [52] for evaluating the effect of obesity depending on menopausal status. However, we stratified participants into pre- and post-menopausal groups when evaluating the influence of obesity clusters on protein levels, but did not identify any proteins with significantly different effects between the strata. Another aspect regarding the analysed population, this study focused on individuals with European ancestry to ensure a large sample size while avoiding population stratification bias. More research and research initiatives focusing on diverse ethnicities are needed to be able to generate findings that are representative of broader parts of the general population [53].

In conclusion, leveraging MR for clustering and two-step mediation analysis we identified four clusters of genetic variants that increase obesity while having differential effects on breast cancer risk. One group of variants had no apparent effect on breast cancer, one increased the risk for breast cancer, another group conferred a medium protective effect, while another cluster appeared to be highly protective. Subsequent mediation analysis identified at least three proteins whose expression varies depending on the cluster. These results hint at the existence of multiple pathways in the development of breast cancer and provide a starting point to explain the complexity of the obesity-breast cancer relationship.

## Supplementary Information

Below is the link to the electronic supplementary material.

Supplementary Material 1
Supplementary Table S1. Information on SNPs included in the MR-clust and the results from the clustering.
Supplementary Table S2. Mendelian randomisation results for the effects of the three non-null clusters on protein measurements.
Supplementary Table S3. Mendelian randomisation results for the effects of the protein measurements on risk of any type of breast cancer and stratified by ER status.

## Data Availability

BMI summary statistics from FinnGen are available upon application under https://www.finngen.fi/en/access_results (file: summary_stats_finngen_R9_BMI_IRN.gz). BMI summary statistics from the GIANT consortium can be downloaded under https://portals.broadinstitute.org/collaboration/giant/index.php/GIANT_consortium_data_files#GWAS_Anthropometric_2015_BMI_Summary_Statistics (file:SNP_gwas_mc_merge_nogc.tbl.uniq.gz), breast cancer summary statistics from the BCAC are available from https://pmc.ncbi.nlm.nih.gov/articles/PMC7808397/, summary statistics for the ER-positive and ER-negative breast cancer are available from https://www.nature.com/articles/nature24284, and protein summary statistics from the UKB-PPP cohort are available under https://metabolomips.org/ukbbpgwas/. UK Biobank data can be accessed upon relevant application to the Biobank itself under https://ukbiobank.ac.uk/.

## Abbreviations

BCAC: Breast cancer association consortium
BMI: Body mass index
CHIAG: Community health index advisory group
CI: Confidence interval
CPM: Carboxypeptidase M
CST6: Cystatin M
ER: Oestrogen receptor
FDR: False discovery rate
GIANT: Genetic investigation of anthropometric traits
GWAS: Genome wide association studies
HGF: Hepatocyte growth factor
IV: Instrumental variable
IVW: Inverse-variance weighted
KELA: Social insurance institution
MET: Hepatocyte growth factor receptor
MR: Mendelian randomisation
MREC: Multi-centre research ethics committee
NAISS: National academic infrastructure for supercomputing in Sweden
NIGB: National information governance board for health and social care
OR: Odds ratio
PGS: Polygenic score
PIAG: Patient information advisory group
REC: Research ethics committee
RTB: Research tissue bank
SNP: Single nucleotide polymorphism
THL: Finnish institute for health and welfare
UKB-PPP: UK biobank pharma proteomics project
